# Strategies for sustainable lung cancer screening: a multi-faceted perspective to long-term surveillance in SOLACE

**DOI:** 10.1186/s13244-026-02259-8

**Published:** 2026-04-07

**Authors:** Emily Nischwitz, Roberta Eufrasia Ledda, Tom Stargardt, Carsten Wältner, Katarzyna Sejbuk-Rozbicka, Joanna Bidzinska, Joanna Moes-Sosnowska, Joanna Chorostowska-Wynimko, Mathis Konrad, Elizabeth Tong, Małgorzata Gałazka-Sobotka, Edyta Szurowska, Dominik R. Dziurda, Hans-Ulrich Kauczor, Viktoria Palm

**Affiliations:** 1 Department of Diagnostic and Interventional Radiology (DIR), Heidelberg University Hospital, Heidelberg, Germany; 2 Department of Medicine and Surgery, University of Parma, Parma, Italy; 3 Fondazione IRCCS Istituto Nazionale dei Tumori of Milan, Milan, Italy; 4 Hamburg Center for Health Economics (HCHE), University of Hamburg, Hamburg, Germany; 5 Lazarski University, Warsaw, Poland; 6 Second Department of Radiology, Medical University of Gdansk, Gdańsk, Poland; 7 Department of Radiology, University Clinical Centre, Gdansk, Poland; 8 Department of Genetics and Clinical Immunology, National Institute of Tuberculosis and Lung Diseases, Warsaw, Poland; 9 Translational Lung Research Center Heidelberg (TLRC), German Center for Lung Research (DZL), Heidelberg, Germany; 10 Diagnostic and Interventional Radiology with Nuclear Medicine, Thoraxklinik Heidelberg, Heidelberg, Germany

**Keywords:** Lung cancer screening, Sustainability, SOLACE, Ecological sustainability, Economic sustainability

## Abstract

**Abstract:**

Lung cancer is the leading cause of cancer-related deaths globally. To detect lung cancer at an earlier, treatable stage, low-dose chest CT is a critical tool for risk-based lung cancer screening. With the growing large-scale, multidisciplinary support for the implementation of lung cancer screening, it is important to ensure these programmes are implemented in a sustainable and effective manner. This review focuses on the three fundamental dimensions of sustainability: ecological, social, and economic. It examines strategies for their effective implementation in accordance with current best practices. Ecological sustainability involves reducing energy use and emissions and leveraging technological innovations to positively impact the environment. Economic sustainability highlights the need to evaluate long-term financial capacity and infrastructure and the role of effective risk-stratification. Social sustainability centres on equitable access to lung cancer screening programmes, particularly for currently underserved populations, with a focus on targeted recruitment strategies. We present an overview of current challenges, an analysis of best practices, and examples of real-world implementation, with a particular emphasis on initiatives in the SOLACE project.

**Clinical relevance statement:**

This review highlights the importance of a multidisciplinary and sustainable approach to implementing lung cancer screening by integrating ecological, economic and social sustainability practices.

**Key Points:**

- Lung cancer screening requires balanced consideration of all three sustainability pillars: ecological, economic, and social.
- This review examines sustainability practices in lung cancer screening, highlighting use cases from SOLACE and beyond.
- Lung cancer screening must embed all aspects of sustainability to achieve lasting clinical and social impact.

**Graphical Abstract:**

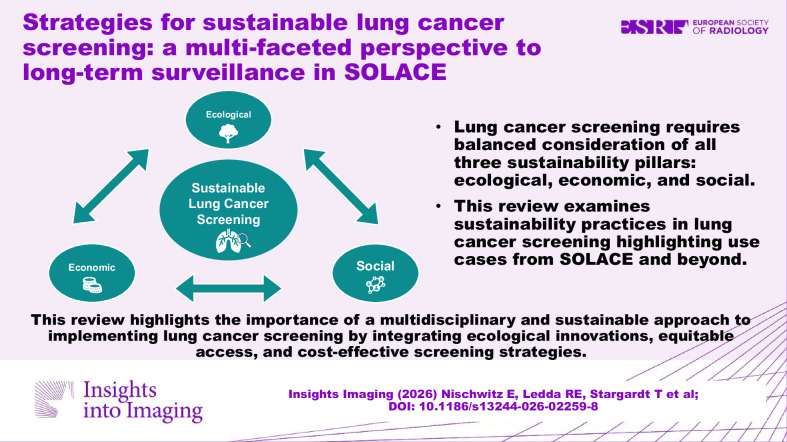

## Introduction

Lung cancer screening (LCS) using low-dose chest computed tomography (LDCT) is an essential tool in combating the highest mortality cancer worldwide [1, 2]. Major clinical trials have shown that LCS reduces mortality by at least 20% in high-risk individuals [3–5]. Yet, despite wide multidisciplinary support, there is limited national implementation of LCS in EU member states [1, 6]. Recognising this gap, the European Commission channelled funds towards Strengthening the Screening of Lung Cancer in Europe (SOLACE), an EU-wide implementation project, which aims to drive the adoption of equitable LCS across Europe [7]. This joint initiative, led by the European Society of Radiologists and the European Respiratory Society, unites 37 partners from 15 countries. These partners include experts from the fields of radiology, pulmonology, thoracic surgery, primary care and family medicine, health economics, medical physics, and healthcare data analytics, among others. As part of this project, there are 19 pilots prospectively recruiting lung cancer screening participants previously underrepresented in LCS, including women, hard-to-reach individuals, and individuals who have comorbidities associated with a higher risk of lung cancer. As this initiative advances, it raises a pressing question: can equitable LCS be implemented while maintaining ecological, economic, and social sustainability?

These three pillars of sustainability are essential to achieving truly sustainable LCS throughout the entire lung screening process, from recruitment to the delivery of medical care [8–10].

Ecological sustainability in healthcare and LCS emphasises the adoption of practices that reduce environmental harm and promote long-term ecological balance [11]. The global environmental footprint of all sectors has continued to grow over time [12]. The healthcare field accounts for an average of 5.5% of the total carbon footprint of a nation, only behind energy, transportation, and construction industries [13]. Energy consumption and emission reduction have come to the forefront of the radiology field, where imaging technologies like LDCT, the primary method for LCS, are inherently energy-intensive [14, 15].

Economic sustainability involves ensuring that financial resources are adequate not only for the short term but also for the continued feasibility of initiatives in the future. The long-term cost-effectiveness of LCS is a key consideration in its implementation. A systematic review analysing all full economic evaluations of LCS between 1994 and 2022 found that 86.7% of studies concluded LDCT LCS to be cost-effective in high-risk groups [16]. In Europe, studies conducted in Germany, Switzerland, and the Netherlands unanimously supported LDCT as a cost-effective screening method when considering the additional quality of life years gained [17–22]. To ensure long-term access to LCS, its financial viability must be continuously evaluated in the context of evolving healthcare environments.

Social sustainability in healthcare focuses on ensuring that all members of the community have access to life-improving medical services. Until this point, LCS programmes exhibit a significant underrepresentation of women, ethnic minorities, socioeconomically disadvantaged groups, individuals living in remote areas, and those with lung-related comorbidities [7, 23–26]. To achieve truly equitable care, it is imperative that all individuals have access to these programmes.

This narrative review highlights best practices identified in the literature and actively employed at SOLACE LCS sites to promote equitable implementation while ensuring ecological, economic, and social sustainability.

### Ecological sustainability

#### Energy consumption and emission reduction

A life-cycle footprint analysis from 33 hospitals in Switzerland identified electricity (12%), building infrastructure (15%), food/catering (17%), and heating, ventilation and air conditioning (HVAC) management (26%) as the most significant contributors to CO_2_-equivalent emissions in healthcare [27]. Whereas, according to a German-wide survey on the importance of sustainability in Radiology, the most critical factors for achieving a more sustainable radiology are waste management (22.6%), energy reduction (16.5%), fostering conscious behaviours (13%), and minimising obsolete or redundant examinations (12.2%) [28]. Optimising these climate change contributing factors is not just a key environmental consideration for an ecologically sustainable LCS. Given the rising lung cancer rates influenced by environmental factors like air pollution, adopting sustainable actions such as “Reduce, Reuse, Recycle” and eco-friendly technologies in screening programmes is crucial for a long-term public health strategy [29].

One of the most significant factors in LCS-related energy consumption is the CT scanner. A typical chest CT scan consumes approximately 1.03–2.2 kWh per examination [30]. However, Tobias Heye et al evaluated that an additional two-thirds of energy consumption was caused by the non-productive, idle state [31]. The total annual energy use for all CT examinations is 3580 kWh, which is comparable to what a two-person household in Switzerland uses in a year. Similar to CT scanners, studies indicate that the standby mode of many Picture Archiving and Communication System (PACS) workstations is inefficient, consuming nearly as much energy as when the devices are in active use [31]. Büttner et al assessed that the consequent turning off of all 227 PACS workstations at the Charité Berlin after core working hours reduces annual CO_2_ emissions by 22.2 tons, representing a 38.6% decrease. This highlights the importance of raising awareness about switching off devices, implementing automated shut-off systems, and advancing technologies that enhance energy efficiency in standby and idle states to reduce LCS-related energy consumption [14, 30, 32].

Industrial manufacturing processes are the second largest sector contributing to the ecological footprint worldwide, accounting for 29% of global CO_2_-equivalent emissions across all economic sectors [33]. Driven by political regulations, manufacturers are aiming for climate neutrality through optimised infrastructure and transportation, by larger, less frequent production batches to reduce the carbon footprint per CT scanner [34]. Renewable energy use, refurbishment processes, and manufacturing-related emissions should further be incorporated into purchasing decisions. Further, transitioning to electronic medical records reduces paper waste, supports data accessibility and security, and promotes streamlined healthcare delivery across sites. As paper accounts for approximately 35% [35] of municipal solid waste, digital systems can reduce environmental impact. Nevertheless, digital systems emit about 0.361 kg of CO₂ per patient visit, nearly ten times the emissions associated with paper records (0.037 kg CO₂ per visit). Hence, renewable energy is a key factor in mitigating these environmental challenges [36]. Across all SOLACE sites, electronic documentation is being used to further reduce paper waste.

Within SOLACE, sites have particularly had an impact on lowering their carbon footprint by utilising mobile CT trucks. These trucks travel to remote communities allowing for far less travel by the screening participants. The majority of these trucks are also equipped with smoking cessation and spirometry services, thus again allowing for both healthcare personnel and screening participants to travel less in order to administer and obtain the necessary services.

#### Technological innovations and best practices

LDCT recommendations for LCS focus on reducing radiation exposure while maintaining diagnostic efficacy. Hence, recent guidelines have reduced the recommended volume computed tomography dose index (CTDI_vol_) by more than half, from 3 mGy [37] to 0.4–1.6 mGy, depending on weight [38, 39]. Deep-learning (DL) networks and artificial intelligence (AI) can further optimise imaging protocols in LCS, ensuring high-quality imaging while minimising exposure. Thereby, DL image reconstruction has demonstrated a reduction in radiation doses by 75% [40] compared to traditional LDCT.

AI-driven workflows have further shown to support automating routine tasks, triaging cases, and flagging abnormalities, and thereby reducing resource consumption [29, 41, 42]. Automated imaging analyses assessing lung nodules enhance the diagnostic accuracy of lung cancer, with a sensitivity of 93.2% [43] and a false positive rate of 0.44 per scan. Besides showing improved reader performance [44], deep-learning computer-aided detection (DL-CAD) software has been shown to reduce radiologists’ reading time for chest CTs by up to 62% [45, 46] and thereby significantly improve workflow efficiency. Specifically, under the rising demand for LCS, integrating AI in LCS can optimise the management of high-risk populations, monitor disease progression, wider integration of systematic evaluation of incidental findings, and personalise follow-up schedules. Within SOLACE, more than 75% of sites involve a primary or secondary AI reader within their screening workflows.

The convergence of technological innovation and sustainability in LCS practices provides a dual benefit: improving health outcomes and reducing the environmental footprint of healthcare services.

### Economic sustainability

Many countries mandate that healthcare interventions demonstrate cost-effectiveness before they can be considered for implementation [47]. In the context of public health investment, LDCT screening programmes should not only contribute to sustainable improved population health outcomes but also ensure equity in access [48]. Establishing the economic justification for large-scale investment in LDCT screening programmes remains a critical consideration.

#### Financial capabilities

In order for LCS to be offered on a regular basis by (public) healthcare systems, continuous funding for such programmes would need to be available on a long-term basis. With the clinical benefits of LCS being undisputed and the cost-effectiveness of LCS being (mostly) within acceptable national ranges for decision-makers [16, 49], the large number of those potentially eligible for LCS constitutes a challenge for many healthcare systems.

Goulart et al estimated that LCS would add $1.3 billion ($2.0 billion) at 50% (75%) participation rate to the US healthcare expenditure in 2011, constituting a 12% (19%) increase in the cost for lung cancer [50]. Similar increases could be calculated for Germany, as Hofer et al [18] estimated a budget impact of €1.84 billion over 15 years from 2016 onwards, given a participation rate among individuals with heavy tobacco consumption of 54% and an annual economic burden for lung cancer of about €2.0 billion [51]. SOLACE has added information on budget impact, in particular for eastern, southern and central European countries, i.e., Croatia, Estonia, and Poland.

#### Capacity and infrastructure

Beyond financial considerations, the successful implementation of LCS programmes necessitates an assessment of the healthcare system capacity and infrastructure. Policies should be tailored to the country/region and based on evidence in the related health system. While considerations should include all aspects throughout the lung cancer care pathway, there should be a particular emphasis on workforce and technical capacity [52].

Determining the availability and deployment of CT scanners in the region of interest is crucial for strategic planning and feasibility. This will enable fair and equal access for residents of the entire region. Analysis and planning components should include the type of database, data collection methods, evaluation, archiving, and appropriate quality assurance measures. A mindful integration of standardised data collection infrastructures enhances research capabilities to facilitate continuous improvements to LCS workflows and streamlines quality assurance processes.

Quality assurance is a cornerstone of sustainable LCS programmes, ensuring both clinical effectiveness and efficient resource utilisation. As highlighted by Rydzak et al [53], integrating robust quality assurance procedures and potentially quantitative imaging biomarkers throughout the LDCT screening process is essential for optimising outcomes and minimising unnecessary costs. Quality assurance by standardised LCS training in image interpretation is essential. Additionally, recent work underscores that standardising CT acquisition protocols not only reduces radiation exposure but also provides a framework for harmonisation across centres, facilitating protocol optimisation and potentially lowering costs related to technical variability and repeat imaging [54]. By promoting cross-continental collaboration and protocol consistency, these efforts directly contribute to the financial and operational sustainability of LCS initiatives.

As LCS programmes increase the detection of early-stage malignancies, demand for surgical interventions is expected to rise, as early-stage lung cancer is primarily treated by surgical resection, whereas advanced disease is managed with radiotherapy and chemotherapy. Modelling studies conducted in the United States indicate that achieving an adherence rate of 50% would necessitate a 37% expansion in surgical capacity [55]. Further, the increasing demand for molecularly targeted therapies highlights challenges in treatment accessibility, as access in some Central European countries, including Poland, remains limited to patients within national drug programmes. Strategies for equitable access to LCS and lung cancer care are therefore key concerns for a sustainable medical infrastructure [56].

A phased LCS implementation strategy, informed by predictive modelling, is recommended to ensure the optimal distribution of healthcare resources. This allows for the identification of areas for development, assessment of the costs of screening and necessary interventions, and allocation of resources according to treatment needs. Also clarifying if the existing technical infrastructure and workforce would best adapt to a centralised, decentralised, or hybrid model [52]. Resource needs are highly context-dependent and will vary across healthcare systems, underscoring the importance of evaluating these requirements individually within each programme.

#### Risk stratification

Given the current low growth in GDP (Gross Domestic Product) that limits the generation of additional tax-based or contribution-based funding for healthcare systems and the reluctance of policymakers to shift more resources into health from publicly funded activity, risk-stratification/prioritisation among those currently considered eligible for LCS programmes could be an option to start implementing LCS on a sustainable base. For example, starting programmes on a lower scale, i.e., for those that would benefit most, could help ensure a lower budget impact and allow the development of sustainable organisational structures and infrastructure that would make a later expansion of LCS easier.

Risk-based screening has been identified as a potentially effective strategy to enhance the equilibrium between the benefits and drawbacks of current population-based cancer screening programmes. Unlike traditional screening models, which adopt a universal, one-size-fits-all approach, risk-tailored screening adapts key aspects, such as eligibility criteria, testing frequency, intervals, and test modalities, according to the individual’s specific risk profile [57].

However, the adoption of risk-adjusted cancer screening at the population level presents significant challenges, as it necessitates modifications across various tiers of practice, including those at the individual, healthcare provider, and healthcare system levels. This is clearly illustrated in the diverse inclusion criteria which are being utilised across the LCS sites in SOLACE. While all sites consider age and previous smoking history, one-third of sites consider more risk-tailored inclusion criteria, which especially take smoking-related pre-existing conditions, such as emphysema, Chronic Obstructive Pulmonary Disease (COPD), and previous cancer history [7].

### Social sustainability

In a healthcare context, social sustainability involves the capability of healthcare services to improve the quality of life and overall well-being of the entire population. It also encompasses the healthcare system’s responsibility to ensure equity and fairness in access to healthcare services for all individuals [58]. Regardless of the type of cancer, the promotion of oncological screening programmes advances the quality of life in the general population, but much still needs to be done to reduce social and economic inequalities [59, 60].

#### Participants, recruitment, and enrolment

Recruitment and enrolment of eligible individuals for LCS programmes remains a multi-faceted challenge [61–63]. In the US, as little as 16% of eligible individuals are participating in LCS [64]. In England, between 20 and 50% of eligible individuals have participated in LCS [65, 66]. These relatively low participation rates are one of the major reasons that the SOLACE project implemented recruitment-focused pilots targeting previously marginalised groups.

Although LCS has not yet become a standard of care in Europe, several pilot studies conducted in both European and non-European countries have shown some disparities in access to LCS services among subjects with different backgrounds [62]. Namely, individuals with higher socioeconomic status appear to be overrepresented in LCS programmes, as compared with those with more deprived backgrounds [67, 68]. Also, ethnic minorities and women tend to be underrepresented in some LCS programmes, even though the benefit of LCS seems greater for female subjects [5, 69, 70]. Notably, subjects with lower socioeconomic status are more likely to experience difficulties related to logistics (e.g., inflexible work hours, travel to and from the LCS centre), while ethnic minorities might face communication-related problems (e.g., language barriers), thus limiting their adherence to LCS services [26, 71].

With the aim of addressing such inequalities, several strategies for outreach and education have been put in place across different countries [23, 25, 26, 72]. These include the use of mobile CT in both rural and urban areas, to facilitate access to chest LDCT scans, demonstrating an increased participation of subjects from geographically remote regions as well as those with lower income [73–75]. This strategy has the potential to allow for greater engagement from high-risk populations and to reduce energy consumption, compared to traditional hospital-based screening, specifically in the countryside. By bringing the screening closer to the patient population, these mobile CT units can improve accessibility and decrease travel-related emissions at the same time.

Other strategies rely on the involvement of general practitioners (GPs) [21, 76] and health personnel dedicated to LCS [68]. In a meta-analysis of 36 studies, Lin et al extrapolated that participants were 2.6 times more likely to participate in LCS programmes when recommended by a healthcare professional [63, 77]. GPs are supporting recruitment across multiple LCS sites in SOLACE, including the national LCS programme in Croatia and Czechia, as well as in Estonia, in the Lung Health Check programme in Ireland and Poland [21, 78–81]. For example, in Estonia, the systematic and personalised contact to the population achieved an exceptional participation rate of 79.3% [81].

More recently, community-based recruitment has been proposed as a valid alternative in the setting of oncological screenings, including LCS [74]. This approach involves individuals who do not necessarily have a medical background but have a deep knowledge of the community where LCS adherence needs improvement. It is thought that in every community there are potential “natural helpers” to be recruited and trained to enhance their innate abilities and knowledge about health issues, who can also be represented by subjects who have gone through oncological diagnoses and treatments [82].

In SOLACE, where a key objective is to improve the recruitment of vulnerable and marginalised communities, often described as “hard to reach”, significant emphasis has been placed on the role of “natural helpers”, also known as ambassadors [7]. These ambassadors have been identified to contact potential participants and increase their adherence to the programme. SOLACE designed a targeted programme to train these individuals on the importance of LCS and how to best disseminate that message [82]. Also, to reach out to ethnic minorities and address language barriers, ambassadors specifically trained in health promotion and risk reduction, and dedicated “ambassadors” enrolled from those being screened, have been trained to contact potential participants and increase their adherence to the programme. Multiple programmes within SOLACE are utilising these ambassadors, creating a unique opportunity to compare enrolment rates of the vulnerable and marginalised with those of the overall population.

#### Smoking cessation and general health awareness

LCS mostly relies on age and smoking status, with smoking cessation remaining the most effective behaviour change to reduce LC-related mortality [83–85]. In line with the most recent guidelines released across Europe and the US, smoking cessation interventions should be incorporated into LCS. The majority of sites involved in SOLACE provide smoking cessation. Some authors have underlined that LCS attendance may provide an excellent opportunity for smoking cessation intervention because LCS participants tend to be overall more concerned with their general health [86–88]. Of note, some research suggests that LCS represents a “teachable moment” for smoking cessation, whereby people with active tobacco use might be particularly receptive to smoking cessation interventions [89–91]. Moreover, it has been shown that the involvement of dedicated personnel, such as tobacco cessation nurse specialists and/or pulmonologists, seems to be more effective and economically sustainable when it comes to smoking cessation strategies [92, 93]. As such, LCS programmes can be utilised to enhance general health awareness amongst screenees, which may have a positive impact on smoking cessation rates as a secondary prevention of lung cancer. This may additionally heighten awareness of the increased risk for pulmonary and cardiovascular diseases, as a result of tobacco smoking [94, 95].

## Conclusion

Establishing long-term lung cancer surveillance through screening programmes requires a multi-faceted strategy that integrates sustainable practices across ecological, social, and economic aspects.

Momentum for the widespread implementation of LCS has never been stronger, making it more urgent than ever to integrate sustainable practices into these programmes from the outset. As LCS programmes continue to grow, policymakers and health authorities have both the opportunity and responsibility to implement programmes that reduce environmental harm, possess long-term economic viability, and prioritise access to all communities. Within routine patient care, healthcare professionals will serve a key role in practically putting these policies and programmes into the clinic. The SOLACE project offers promising examples of how sustainable practices can be translated into real-world implementation, guiding future efforts across Europe and beyond.

## Data Availability

Data available upon request.

## Abbreviations

AI: Artificial intelligence
DL: Deep-learning
GPs: General practitioners
LCS: Lung cancer screening
LDCT: Low-dose computed tomography
PACS: Picture archiving and communication system
SOLACE: Strengthening the screening of lung cancer in Europe
